# Supplementary material for: Predicting visual function by interpreting a neuronal wiring diagram
Source: Nature. 2024 Oct 2;634(8032):113–23. doi: 10.1038/s41586-024-07953-5 (PMC11446822; doi:10.1038/s41586-024-07953-5)
Supplement: Supplementary file 6 — CRF and ERF predictions for individual TmY4 and TmY9 cells. Analogous to Supplementary Data 3, but for TmY target types. Shown are the top four monosynaptic pathways, the strongest pathway passing through each of the top ten intermediary types (ranking from Extended Data Fig. 7), and the trisynaptic pathway Tm1–TmY–Dm3–TmY (see the section entitled Prediction of spatial normalization). [file 41586_2024_7953_MOESM6_ESM.zip › DataS4/TmY9qΓèÑ/720575940638828614.pdf]

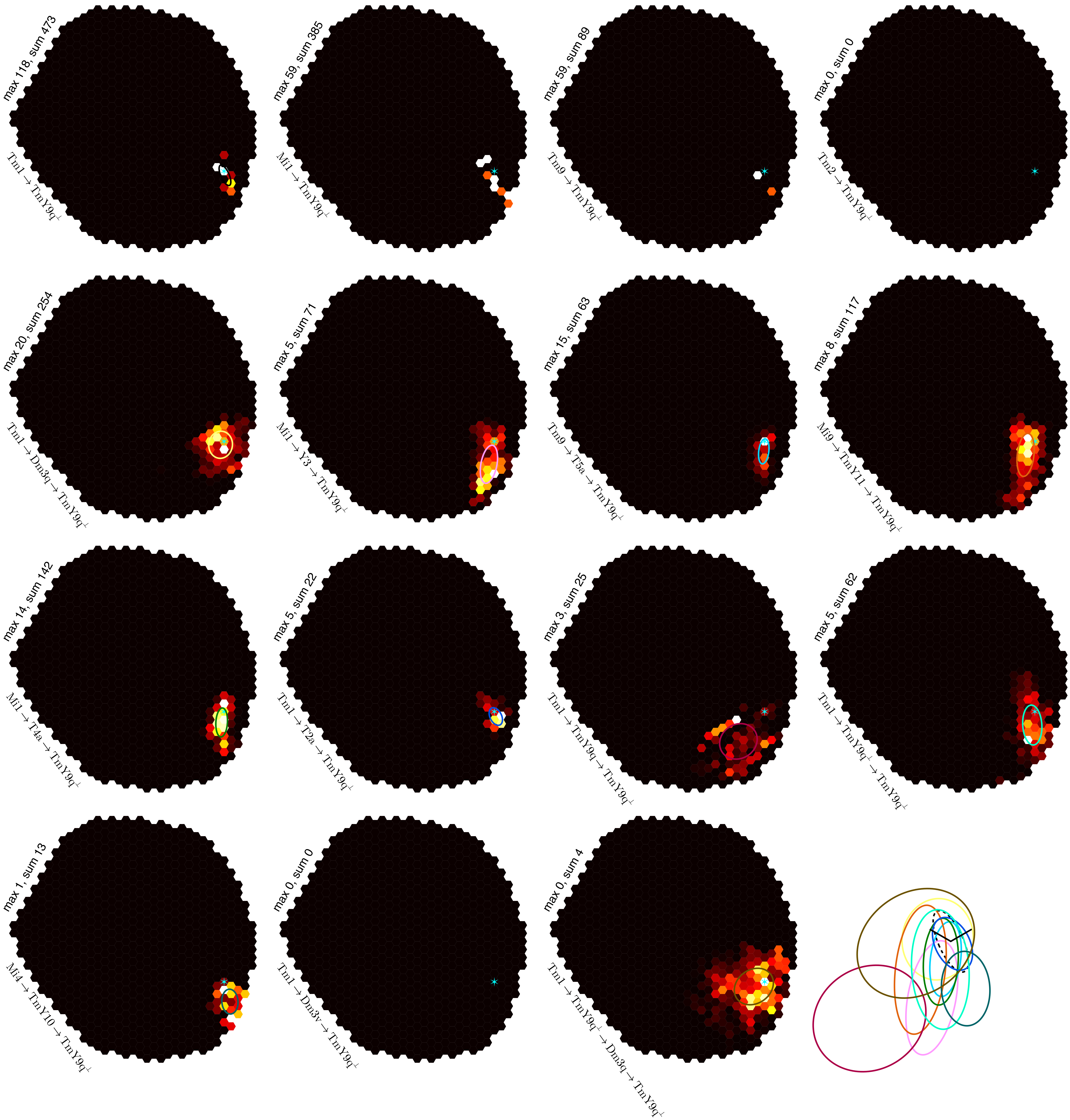

max 118, sum 473
Tm1 → TmY9q⊥
max 59, sum 385
Mi1 → TmY9q⊥
max 59, sum 89
Tm9 → TmY9q⊥
max 0, sum 0
Tm2 → TmY9q⊥
max 20, sum 254
Tm1 → Dm3q → TmY9q⊥
max 5, sum 71
Mi1 → Y3 → TmY9q⊥
max 15, sum 63
Tm9 → T5a → TmY9q⊥
max 8, sum 117
Mi9 → TmY11 → TmY9q⊥
max 14, sum 142
Mi1 → T4a → TmY9q⊥
max 5, sum 22
Tm1 → T2a → TmY9q⊥
max 3, sum 25
Tm1 → TmY9q → TmY9q⊥
max 5, sum 62
Tm1 → TmY9q⊥ → TmY9q⊥
max 1, sum 13
Mi4 → TmY10 → TmY9q⊥
max 0, sum 0
Tm1 → Dm3v → TmY9q⊥
max 0, sum 4
Tm1 → TmY9q⊥ → Dm3q → TmY9q⊥
